# Polyphenolic and Immunometric Profiling of Wheat Varieties: Impact of Organic and Conventional Farming on Allergenic and Bioactive Compounds

**DOI:** 10.3390/molecules30061313

**Published:** 2025-03-14

**Authors:** Adrian Bartos, Alicja Malik, Beata Feledyn-Szewczyk, Krzysztof Jończyk, Renata Kazimierczak, Ewelina Hallmann, Joanna Leszczyńska

**Affiliations:** 1 Department of Bioinorganic Chemistry, Faculty of Pharmacy, Medical University of Lodz, Jana Muszyńskiego 1, 90-151 Łódź, Poland; 2 Institute of Natural Products and Cosmetics, Faculty of Biotechnology and Food Sciences, Łódź University of Technology, Stefanowskiego 2/22, 90-537 Łódź, Poland; alicja.malik12@gmail.com; 3 Department of Agroecology and Economics, Institute of Soil Science and Plant Cultivation, Czartoryskich 8, 24-100 Puławy, Poland; bszewczyk@iung.pulawy.pl (B.F.-S.); kjonczyk@iung.pulawy.pl (K.J.); 4 Department of Functional and Organic Food, Institute of Human Nutrition Sciences, Warsaw University of Life Sciences, Nowoursynowska 159c, 02-776 Warsaw, Poland; renata_kazimierczak@sggw.edu.pl (R.K.); ewelina_hallmann@sggw.edu.pl (E.H.); 5 Bioeconomy Research Institute, Agriculture Academy, Vytautas Magnus University, Donelaicio 58, 44248 Kaunas, Lithuania

**Keywords:** wheat, gluten, profilin, polyphenols, allergy, agriculture

## Abstract

This study investigates the impact of organic and conventional farming on the allergenic and bioactive properties of wheat. The primary aim was to assess the immunometric parameters and polyphenolic composition in four varieties of winter and four varieties of spring wheat cultivated under both farming systems. Immunometric assays focused on gluten content, the allergenic QQQPP peptide, and the panallergenic profilin Tri a 12. While gluten levels (15–20 g/kg) showed no clear dependence on farming type, organic wheat exhibited a mild yet significant increase in QQQPP-dependent immunoreactivity in five samples (>20 µg/g). However, all organic wheat samples demonstrated a notable reduction in profilin content (<0.6 µg/g), suggesting that the type of wheat cultivation could influence allergenic risk for individuals with wheat-related allergies. Polyphenolic profiling revealed that kaempferol, p-coumaric acid, and gallic acid were the predominant compounds, with organic wheat displaying slightly higher polyphenol levels on average. Despite these differences, the variations were insufficient to determine a superior cultivation method. These findings highlight the potential allergenic and nutritional implications of organic versus conventional wheat farming.

## 1. Introduction

For the last few decades, farmers have been relying on the innovation brought by synthetic chemical fertilizers and pesticides used for the benefit of agricultural produce [1]. The former are applied to enhance crop production. Fertilizers contributed up to a 50% increase in 20th-century crop yields, with rice yields rising by 19–41% and rapeseed by 61–76% [2]. At the same time, though, fertilizers are known to introduce a load of acidic chemicals [3] and poisonous toxins into the soil [4], diminish its organic carbon pool [5], disturb the microbial biomass structure [6], harm subterranean organisms [7], cause ammonia emissions to the atmosphere [8], leach into and contaminate underground and surface water reservoirs [9]. Pesticides are crucial in protecting crops from weeds and insects, preventing yield losses of 78% in fruits, 54% in vegetables, and 32% in cereals [10]. The misuse or overuse of pesticides harms the environment, leading to bioaccumulation in aquatic animals and persistence in the food chain until human consumption [11]. The retention time of some of the pesticides in the ecosystem is long, and chronic exposure to them is a severe health hazard [12]. Also, more and more species develop resistance against pesticides [13,14], which brings their further use into question. The impact of both fertilizers and pesticides is therefore more ambiguous than what was originally intended for them [15,16]. After years of harmful practices, farmers are increasingly realizing that quick fixes have become problems themselves [17].

In response to these concerns, organic farming was introduced as an agricultural trend that aims to reduce environmental burden on cultivated terrains [18] and views the entire ecosystem as sustainable, clean, and free from xenobiotics [19]. It abandons fertilizers, pesticides, fossil fuels, and, overall, any synthetic means of interference that would modify the natural flora and fauna. Although organic practice is generally considered to yield more perishable [20] and more variable crop output [21], compared to conventional farming, it was also shown to favorably affect biotic abundance, biotic richness, soil carbon, and profitability [21].

Conflicting data exist on the superiority of organic crops in terms of biochemical and bioactive components. Most sources agree on organic produce containing smaller amounts of nitrate, nitrite, and pesticide residues; antibiotics; food additives; industrial pollutants and heavy metals, and higher or equal amounts of mineral elements; vitamins; secondary metabolites; phenolic compounds; antioxidants; anthocyanins; isoflavones; carotenoids; dry matter; total sugars and antioxidant activity; and higher protein quality [22]. Many factors influencing the health benefits of organic produce remain unstudied. Our research aims to fill these gaps by identifying molecular advantages in its composition.

Wheat can pose a potential threat to consumers who suffer from celiac disease or non-celiac gluten/wheat sensitivity. Ingested gluten evokes gastro- and extraintestinal manifestations in genetically susceptible patients. To minimize the risk of health issues, life-long gluten-free elimination diets are advised, which exclude dietary products based on wheat, rye, or barley [23] and include no more than 20 ppm of gluten. Daily consumption of more than 50 mg gluten is a safe, allowable maximum for celiac patients, as opposed to the typical amount of 10–20 g [24]. It is therefore reasonable to monitor the content of gluten in foods and raw materials and to know how given agricultural or processing practices affect its occurrence and levels, ensuring better control over its presence in the food supply.

IgE-mediated allergy is another disorder associated with the consumption of wheat. Found in low molecular weight subunits of glutenin, the QQQPP peptide motif is the main IgE-binding epitope in patients hypersensitive to wheat [25]. Otherwise, an allergic reaction may also occur in response to wheat profilin, known as Tri a 12. The protein largely shares a sequence and structure with other profilins from numerous other plants. Highly conserved profilins are responsible for the same biological processes across different organisms, which warrants their similarity and ubiquity [26]. Wheat profilin is therefore recognized by specific IgE antibodies in patients suffering from baker’s asthma, wheat-induced food allergy, or grass pollen allergy [27]. Given the prevalence of hypersensitivities to the components of wheat, it is also vital to consider the allergenicity of the crop when evaluating the properties of the produce.

In this work, we conducted an immunometric analysis of wheat, focusing on gluten, the QQQPP peptide, and profilin—key factors in gastrointestinal health. Additionally, we examined polyphenolic compounds to assess bioactive variability. Comparing organic and conventional samples, we could conclude on how the type of crop cultivation translates to changes of both allergenic and bioactive compounds in organic wheat production.

## 2. Results

### 2.1. Gluten Quantification

**Figure 1:**
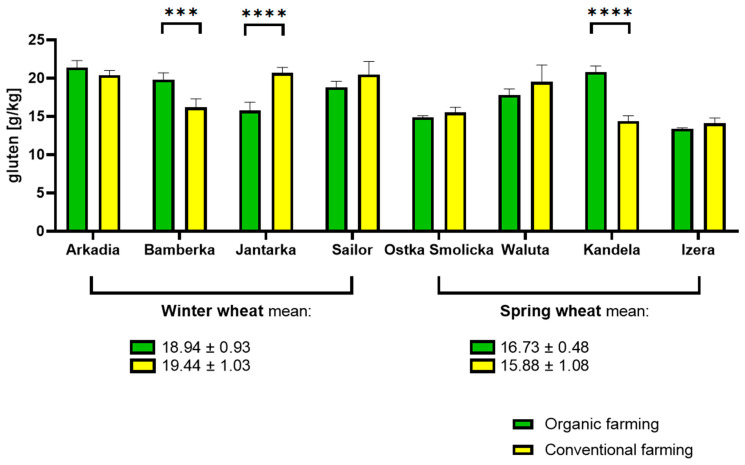
Gluten content determined in winter and spring wheat cultivated on an organic farm and on a conventional farm. (*** adjusted *p* value = 0.0009, **** adjusted *p* value < 0.0001).

The gluten content showed a similar distribution between the respective wheat varieties, ranging from the minimum 13.4 (organic Izera) up to 21.4 g/kg at most (organic Arkadia). No more than a 20% difference was reported in any of the eight pairs of tested samples. Differences reported in only three out of eight pairs of samples were considered statistically significant. Otherwise, the pattern of changes was found to be random. Therefore, no binding conclusions could be drawn about the type of cultivation and its impact on gluten in wheat. Regarding the average, winter varieties were a little more abundant in gluten than spring varieties, whether organic or conventional. The difference between the highest reading reported in winter wheat sample and the lowest reading of gluten content in spring variety sample was approximately 35%. The results are shown in Figure 1.

### 2.2. QQQPP Peptide Determination

**Figure 2:**
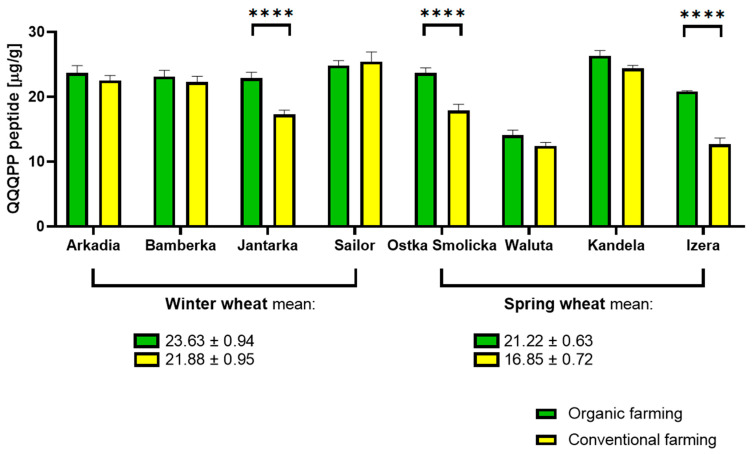
Immunoreactivity, expressed as a content of allergenic QQQPP peptide, determined in winter and spring wheat cultivated on an organic farm and on a conventional farm. (**** adjusted *p* value < 0.0001).

The immunoreactivity of wheat was somewhat sensitive to the type of farming. Wheat cultivated in line with organic farming principles exhibited either comparable or—in most cases—a higher content of allergenic QQQPP peptide than wheat cultivated conventionally. These differences were more pronounced in the spring wheat group than winter wheat, but the magnitude of these changes is mostly moderate. Three of eight tested pairs of samples indicated a statistically significant difference. On average, winter wheat varieties consisted of more antigen than the spring ones. The most QQQPP-abundant winter wheat sample, organic Sailor, at 24.80 exceeded the lowest reading marked for the spring variety, conventional Waluta, at 12.40 µg, by 50%. The highest reading was, however, reported in the conventional Sailor wheat sample, at 25.42 µg QQQPP per gram of sample. The results imply that the wheat farming method could be one of the factors capable of modulating cereal immunoreactivity in the earliest stage of food production, preceding any successive food processing operations on harvested crops. The effect of crop cultivation is, however, not universal and only impacts a minority of tested samples. The results are shown in Figure 2.

### 2.3. Profilin Determination

**Figure 3:**
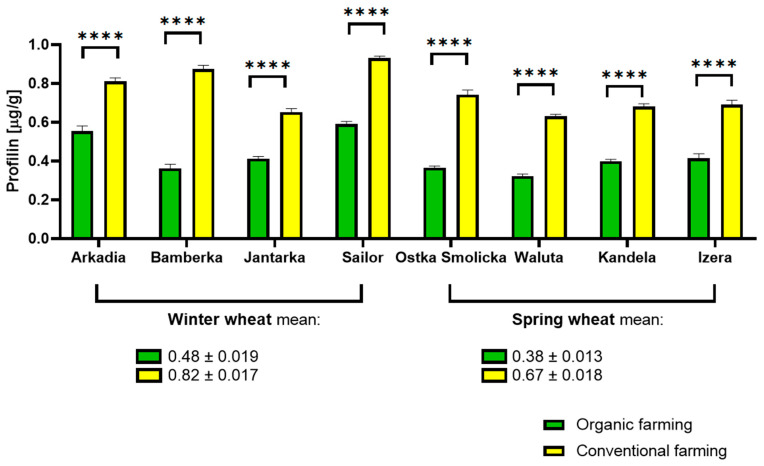
Profilin content determined in winter and spring wheat cultivated on an organic farm and on a conventional farm. (**** adjusted *p* value < 0.0001).

Most notably here, organic farming was shown to be conducive to limiting the content of profilin in all tested samples, whether winter or spring wheat, and regardless of the individual variety of the crops. In this case, the effort associated with organic farming entailed a desired outcome, adding to its practical significance. The impact exerted by the type of cultivation was clear and much more pronounced than differences occurring naturally between distinct varieties, even when comparing winter and spring samples. Crops subjected to organic farming exhibited up to half the profilin content determined in wheat cultivated conventionally. On average, winter wheat exhibited greater profilin content than spring wheat. The decrease observed between the highest winter wheat sample (conventional Sailor, at 0.93 µg/g) and lowest spring wheat sample reading (organic Waluta, at 0.32 µg/g) was nearly 65%. The results are shown in Figure 3.

### 2.4. Analysis of Polyphenols

Table 1 summarizes the overall composition of polyphenolic compounds averaged from eight varieties of each kind of cultivation. Differences between the organic and conventional crops are typically moderate (within 1 mg per 100 g) and possibly too subtle to anticipate pronounced health-promoting effects under normal daily consumption from either of them. It should be noted, though, that with the exception of apigenin and kaempferol, marginally higher amounts of bioactive compounds are consistently reported in wheat grown organically. Gallic acid, p-coumaric acid, and kaempferol were identified as the main components in each sample, while the other polyphenols occurred at up to 5 mg per 100 g. Detailed data on each of the varieties subjected to the test are available in Table 2 and Table 3. When comparing organic and conventional samples of the same variety side by side, the same pattern is reported where organic wheat is marked by a mildly higher content of most of the listed polyphenols, excluding abundant kaempferol.

**Figure 4:**
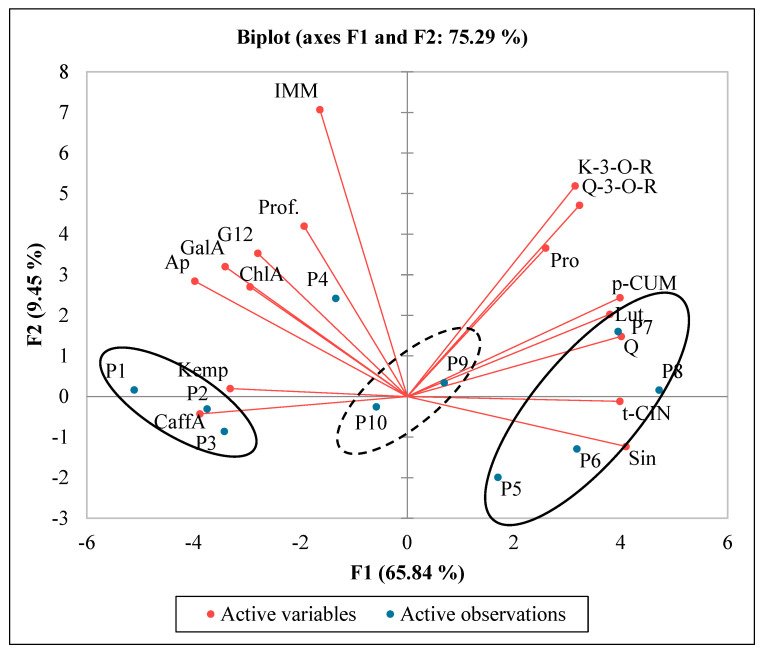
PCA analysis showing the relationship between the chemical composition and allergen factors for different wheat varieties. (Pro) proteins, (G12) peptide toxic for celiac patients, (IMM) immunoreactivity, (prof) profilins, (GalA) gallic acid, (ChlA) chlorogenic acid, (CaffA) caffeic acid, (*p*-CUM) p-coumaric acid, (Sin) sinapic acid, (*t*-CIN) *t*-cinnamic acid, (Q-3-*O*-R) rutinoside-3-*O*-quercetin, (K-3-*O*-R) rutinoside-3-*O*-kaempferol, (Lut) luteolin, (Q) quercetin, (Ap) apigenin, and (Kemp) kaempferol. (P1) Arkadia, (P2) Bamberka, (P3) Jantarka, (P4) Sailor, (P5) Ostka Smolicka, (P6) Waluta, (P7), Kandela, (P8) Izera, (P9) ORG, and (P10) CONV.

For an in-depth analysis on the relationship between the chemical composition of polyphenols and allergens in wheat, a PCA analysis was prepared (Figure 4). Principal Component Analysis shows parameters with a positive correlation when grouped together. Herein, we can deduce that polyphenols do not contribute to the immunoreactivity of the samples. However, the further away the variable is from the origin, the stronger its impact on the model. Although uncorrelated to the content of polyphenols, immunoreactivity as a parameter was placed in the most peripheral region of the graph. Based on the PCA results, the overall degree of variability explained by F1 and F2 was 75.29% for the phenolic composition and allergenic factors in the examined wheat cultivars. This result was confirmed by a strong link between the chemical composition of organic and conventional wheats samples and allergenic status. In the case of winter wheats, we observed that three of the four wheat varieties were located closely to each other. Ostka Smol cv. and Walnuta were similar and located in the same area of the chart. On the other hand, Kndela cv. and Izera cv. created a couple as well. Three of four wheat spring varieties, Arkadia cv., Bamberka cv., and Jantarka cv., create a big group located near the horizontal axis. It is worth pointing out that organic and conventional samples were located on opposite fields of the chart. The observations indicate that the quality of organic wheat samples significantly depended on the content of proteins, p-coumaric acid, luteolin, and quercetin-3-O-rutinoside, as well as the kaempferol-3-O-glucoside concentration in the examined samples. The quality of conventional wheat samples was significantly influenced by the caffeic and kaempferol concentration.

## 3. Discussion

Despite rapid technological advancement made in the first two decades of the XXI century, most principles regarding agricultural practice remain unchanged or are being adapted to today’s opportunities at a relatively slow pace [28]. Organic farming is one of these novelties that were thought of as a response to the demands of a modern consumer [29]. Produce originating from an organic farm not only acquires the organic profile, but they are also a tangible affirmation of a more comprehensible approach embracing the sustainability of the entire ecosystem. Apart from the easily discernible environmental benefits it contributes to, organic farming became more than it was initially intended for. Not only the level of purity, but also nutritional [30] and health-related properties [31,32,33] of fruits and vegetables cultivated in line with organic rules are somewhat different than what is known for their counterparts grown conventionally. While the aspect of purity is beyond question, the impact of an organic diet on human health (allergies, obesity, etc.) still needs more scientific evidence and statistical analyses [34].

In our research, significant differences in protein content between organic and conventional farming were only noted in three of eight tested cultivars, and the pattern of changes was found to be random. In Mitura et al.’s study (2023), wet gluten content values were significantly influenced by cultivar and farming systems (organic vs. conventional vs. integrated) [35]. The significantly highest wet gluten content was a characteristic of the wheat grain of the Serenada cultivar, but in the grain of three other cultivars, this parameter was not significantly differentiated. The wheat cultivar differences in gluten content have also been indicated by other authors. Moreover, researchers observed a significant effect of the farming system on gluten content [36,37]. In Mitura et al.’s (2023) study [35], a significantly higher wet gluten content was reported in spring wheat grain obtained from the conventional system (mean 33.4%) in comparison with the organic system (mean 27.5%) due to the higher level of nitrogen fertilization in the conventional system. Similar relationships were shown for winter wheat in research by Sobolewska and Stankowski [38].

As indicated in this work, the adoption of the organic system is particularly well reflected through the level of immunoreactivity of the crops. Organic wheat may carry a moderately elevated content of allergenic QQQPP peptides of gliadin, but what is much more compelling is that it contains considerably less profilin. This fact could translate to mildly increased exposure to the primary allergen in specific varieties of wheat, but at the same time, a substantially more reduced risk of immune cross-reactivity to the ubiquitous panallergen in the majority of wheat varieties. Profilin is a highly conserved protein, the sequence of which can be found in a plethora of various plants with 70% homology [39]. This is because it serves a fundamental cytoskeletal function associated with the polymerization of actin filaments and monomers, the mechanism of which is shared across multiple species [40]. This common role and structure of the molecule is the reason for its natural ubiquity, which, in turn, largely expands the risk of exposure to the cross-reactive epitope. Profilins are, for example, a reason why the prevalence of pollen allergy is estimated to reach as much as 40% [41]. Up to 70% of cases of respiratory sensitization to profilin lead to development of type II food allergy known as pollen-food syndrome [42]. Bet v 2 from birch pollen is the first and the most researched on profilin. It is responsible for allergic rhinoconjunctivitis and asthma [43]. Wheat profilin is known as Tri a 12. It is recognized by IgE antibodies from patients that suffer from baker’s asthma [44], grass pollen allergy [45], and food allergy to wheat [46].

What was shown in this study is that organic farming has substantial potential for modulating some of the health-related properties of food. In this case, the observed effect translates into a change in biochemical composition of crops that could warrant a lower risk of allergic response. A consistently diminished pool of Tri a 12 profilin in wheat cultivated in line with the principles of organic farming was reported. The mean profilin content in organic produce dropped down to 0.43 µg/g compared with conventional samples, where the total mean value was 0.75 µg/g. To the best of our knowledge, no other study has reported profilin changes between organic and conventional wheat. Other food products were, however, researched, which can be indirectly presented as a point of reference for the evaluation of our results.

The result itself, its magnitude and consistency found along multiple different crop varieties, is somewhat surprising and in contradiction to what had been reported on organic produce in the past. In a 2016 article, tomato profilin (Sola l 1, previously known as Lyc e 1) was determined over the course of three years in five distinct varieties [47]. Its content in vegetable samples was shown to be statistically higher in organic samples. The same was true when fruit samples (apricots) were analyzed [48]. Organic herbs from the *Lamiaceae* (basil, oregano) and *Apiaceae* (cumin, fennel, parsley, anise, coriander) families were also found to be more profilin-abundant [49]. Relatively little to no difference was reported between organic and *conventional* raspberry, depending on the year of harvest [50]. This suggests that the relationship between the type of farming and the output content of profilin may not be as straightforward as initially thought of and could be pertinent to the type of plant (crops vs. fruit), if not dependent on more variables associated with specific agricultural parameters.

In our quantitative study on polyphenols, kaempferol, p-coumaric acid, and gallic acid were identified as the most abundant components in all tested wheat varieties. With a mean of 36.96 ± 2.67–38.16 ± 3.14 mg of kaempferol/100 g of grain, depending on the type of crop cultivation, wheat is a richer source of this compound than broccoli, blueberries, and onions. Kaempferol is known to have a broad bioactive impact and is a subject of interest to numerous researchers. Its properties are antioxidant (confirmed regulation via Bax/Bcl-2, caspase-3 molecules, and vascular endothelial growth factor in ARPE-19 cells), anti-inflammatory (confirmed suppression of the activation of Akt and NF-κ in cardiac fibroblasts, regulation of the production of inflammatory mediators and proteins involved in inflammatory response), anti-diabetic (confirmed by studies on diabetic rodents, where it decreased blood glucose levels, recovered lipid peroxidative markers, raised the level of plasma glucose, and reduced the insulin level), cardioprotective (reduced cardiac hypertrophy), neuroprotective (restored cognitive deficits caused by streptozotocin in animal neuronal models), hepatoprotective (decreased hepatic levels of IL-6, TNF-α, and caspase-3, but reduced the increase of circulatory serum levels of γ-GT, ALT, and AST in acetaminophen-treated rats and enhanced anti-oxidative defense in alcoholic liver injury mice), and anti-cancer and tumor-supressing (modulation of cell signaling molecules, including IQGAP3 or MCF-7; regulation of the cell cycle via Chk2/p21/Cdc2 and Chk2/Cdc25C/Cdc2 pathways; impact on gene expression, including MMP-2, MMP-9, ABCC6, and cMyc), to name just a few examples [51]. P-coumaric acid has been ascribed antioxidant, cardio-, and neuroprotective properties, as well as anti-microbial, analgesic, anxiolytic, anti-inflammatory, and anti-mutagenic effects, to list a few [52]. With a content of 27.22 ± 3.89–29.45 ± 4.09 mg/100 g of grain, wheat is more abundant in p-coumaric acid than apples, oranges, grapefruits, and ginseng [53]. It has been researched in the context of colorectal cancer prevention and treatment [52] as a support against depression, memory impairment [54], and other stress-associated mental disorders [55] and as a polymerized nanocarrier for anticancer drug delivery [56].

Gallic acid is also a highly bioactive molecule [57]. Encapsulated into nanoparticles to bypass setback associated with its low bioavailability and fast systemic clearance, gallic acid exerts cytotoxic effects against human neuroblastoma and other cancer cell lines [58]. Its antioxidant, anti-inflammatory, anti-adipogenic, and apoptotic actions have been shown to promote the treatment of obesity [59]. Gallic acid is also linked to the attempted treatment of neurological diseases and disorders, such as Alzheimer’s and Parkinson’s disease [60].

In a study by other authors, the concentration of phenolic acids in winter wheat grain (except for syringic and salicylic acid), as well as the total phenolic acid content (956 µg/g) and its activity (0.204), was higher in organic grain than in the grain of other tested farming systems (organic > integrated > conventional > wheat monoculture) [61]. A higher content of phenolic acids in the grain from organic production can benefit human health.

Naturally composed of kaempferol, p-coumaric acid, and gallic acid, wheat can be used as a source of natural compounds for novel therapeutic solutions, while its cultivation method and conditions should be further explored in an attempt to maximize the pool of these bioactive molecules.

## 4. Materials and Methods

### 4.1. Sample Collection

This study focused on the analysis of both winter and spring wheat (*Triticum aestivum* L. subsp. *aestivum*) samples, grown in four replications for subsequent evaluation. Four varieties of winter wheat—Arkadia, Bamberka, Jantarka, and Sailor—along with four spring wheat varieties—Ostka Smolicka, Waluta, Kandela, and Izera—were examined. These samples were collected from the Agricultural Research Station of the Institute of Soil Science and Plant Cultivation (IUNG-PIB) in Osiny, located in the Lublin region of Poland (coordinates: N: 51°28′, E: 22°30′). The soil at this site was classified as Haplic Luvisol, with a loamy sand texture and a slightly acidic pH of 5.6 (pHKCl), as well as average phosphorus and potassium content. The soil’s organic matter content was 1.5%.

The research compared two farming systems—organic and conventional. Key differences between the two included crop rotation; fertilization methods; and the approach to weed, pest, and disease control. The organic system avoided the use of mineral fertilizers and chemical herbicides, relying instead on a single application of compost (30 t ha^−1^) before planting potatoes as part of the rotation. In contrast, the conventional system followed a three-year crop rotation (winter oilseed rape, winter wheat, and spring wheat), and used intensive agricultural practices, including high levels of mineral fertilization and chemical plant protection (as detailed in Table 4 and Table 5).

In both farming systems, wheat was cultivated annually, with both winter and spring wheat varieties included. A split-plot design was employed, with four wheat varieties tested across four replications, and the treatments were randomized. Both systems applied traditional ploughing for soil tillage. Each plot measured 30 m^2^ at sowing and 25 m^2^ at harvest. Sowing was conducted according to optimal practices for the region, with a consistent seeding rate of 450 seeds per m^2^, a row spacing of 12 cm, and a planting depth of 3.5 cm.

### 4.2. Gluten Quantification

Sample extraction was performed according to the protocol of the manufacturer of AgraQuant Gluten G12 kit (Romer Labs, Getzersdorf, Austria) and using reagents provided therein. Briefly, weighted portions of 0.5 g analytical sample were mixed with 5 mL of Extraction Solution. Vials were vortexed and left on shaker for 40 min at room temperature. Suspension was centrifuged for 10 min at 5600 RPM. Supernatant was diluted with Dilution Solution.

Gluten quantification was performed with the use of AgraQuant Gluten G12 kit (Romer Labs, Getzersdorf, Austria) based on competitive ELISA method. In brief, 0.5 g of sample was mixed with 5 mL of Extraction Solution and kept on a shaker for 40 min at room temperature. Suspension was centrifuged for 10 min at 5600 rpm, and the supernatant was collected. Upon 1000-fold dilution with Dilution Solution, 300 µL sample was mixed with 300 µL antibodies specific against 33-mer gluten peptide. It was incubated on a shaker for 60 min at room temperature. Standard samples, positive and negative controls were prepared in an analogous manner. Duplicates of 200 µL sample were introduced into the analytical plate and incubated for 60 min at room temperature. Wells were washed 5 times with Wash Solution. Substrate Solution at 100 µL was introduced into each well and incubated for 15 min at room temperature in the dark. Reaction was terminated with 100 µL of Stop Solution added to each well. Absorbance was measured at 450 nm.

### 4.3. Enzyme-Linked Immunosorbent Assay (ELISA) for QQQPP Peptide Determination and Profilin Determination

This study followed a previously established protocol [62], with modifications detailed below.

To begin, sample extracts underwent a 1000-fold dilution before being used to coat a 96-well microplate. Negative control wells were coated with a 3% milk powder solution. The plate was then incubated overnight at 4 °C. The next day, the coating solutions were discarded, and the plate was washed four times using PBS-T. To block unbound sites, a 3% milk powder solution was applied, followed by a 2 h incubation at room temperature. Afterward, the plate was washed four times with PBS-T.

Reagents:

PBS buffer: 0.7551 g of KH_2_PO_4_, 11.466 g of Na_2_HPO_4_, and 18 g of NaCl dissolved in 2 L of distilled water.

Carbonate–bicarbonate buffer

PBS-T buffer: 1 mL of Tween per 1 L of PBS.

3% milk powder solution: 3 g of milk powder dissolved in 1 mL of PBS.

(a) QQQPP Peptide Determination

Weighted portions of 0.5 g analytical sample were mixed with 5 mL of 70% ethanol and left on shaker for 40 min at room temperature. Suspension was centrifuged for 10 min at 5600 RPM. Supernatant was diluted with PBS.

A 100-fold dilution of rabbit sera containing anti-QQQPP peptide antibodies was prepared in PBS. A 100 µL aliquot of this solution was added to each well, and the plate was incubated for 1 h at room temperature before undergoing four washes with PBS-T. Next, a goat-derived anti-rabbit IgG-peroxidase antibody, diluted 1000-fold, was applied at 100 µL per well. The plate was incubated for another hour at room temperature and washed four times with PBS-T. Following this, TMB substrate (100 µL per well) was added and left to react for 30 to 60 min at room temperature. The enzymatic reaction was halted by adding 100 µL of 1 M H_2_SO_4_ to each well. Absorbance readings at 450 nm were recorded using a microplate reader. Results were determined based on a standard curve generated from serially diluted QQQPP peptide.

Reagents:

Rabbit serum containing anti-QQQPP primary antibodies.

Anti-rabbit antibodies: Anti-Rabbit IgG (whole molecule)–peroxidase, goat-derived (Sigma-Aldrich, St. Louis, MO, USA).

TMB substrate: 3,3′,5,5′-Tetramethylbenzidine (TMB) Liquid Substrate System for ELISA (Sigma-Aldrich).

1 M sulfuric acid.

(b) Profilin Determination

Weighted portions of 0.5 g analytical sample were mixed with 5 mL phosphate-buffered saline (PBS) buffer and left on shaker for 40 min at room temperature. Suspension was centrifuged for 10 min at 5600 RPM. Supernatant was diluted with PBS.

For profilin detection, rabbit anti-profilin antibodies were diluted 1000-fold in PBS. Each well received 100 µL of the diluted solution, and the plate was incubated for 1 h at room temperature, followed by four washes with PBS-T. Then, a goat-derived anti-rabbit IgG-alkaline phosphatase antibody (diluted 1000-fold) was applied at 100 µL per well. After another 1 h incubation at room temperature, the plate was washed four times with PBS-T. A 100 µL aliquot of pNPP substrate was added to each well and incubated for 30 to 60 min at room temperature. The reaction was stopped by adding 100 µL of 3 M NaOH. Absorbance at 405 nm was measured using a microplate reader, and results were calculated from a standard curve generated using serially diluted profilin solutions.

Reagents:

Rabbit anti-profilin antibodies: Anti-Profilin 1 (C-terminal) antibody, rabbit-derived (Sigma-Aldrich).

Anti-rabbit antibodies: Anti-Rabbit IgG (whole molecule)–Alkaline Phosphatase, goat-derived (Sigma-Aldrich).

p-Nitrophenyl phosphate (pNPP, para-nitrofenylofosforan, Sigma-Aldrich).

3M sodium hydroxide.

### 4.4. Analysis of Polyphenols

#### 4.4.1. Sample Preparation

The identification and quantification of polyphenols, including flavonols and phenolic acids, were conducted using the HPLC method. A 100 mg portion of the wheat sample was combined with 5 mL of 80% methanol (HPLC-grade) and mixed using a Vortex 326 M (Marki, Poland). Subsequently, the samples underwent ultrasonic extraction for 10 min at 30 °C with a frequency of 5.5 kHz. Following this, the extracts were centrifuged for 10 min at 6000 rpm and 5 °C. The supernatant was then collected into a clean Eppendorf tube and subjected to a second centrifugation at 12,000 rpm for 5 min at 0 °C. Finally, 500 μL of the resulting supernatant was transferred into HPLC vials for analysis.

#### 4.4.2. Separation by HPLC

All polyphenol samples were analyzed using a Synergi Fusion-RP 80i column (250 × 4.60 mm) from Phenomenex (Torrance, CA, USA)). The HPLC analysis was performed with Shimadzu equipment (Chicago, IL, USA), which included two LC-20AD pumps, a CBM-20A controller, an SIL-20AC column oven, and a UV/Vis SPD-20 AV spectrometer (USA Manufacturing Inc., Gaithersburg, MD, USA).

The separation of phenolic compounds, including flavonols and phenolic acids, was conducted under gradient conditions with a flow rate of 1 mL/min. Two mobile phases were utilized: phase A consisted of 10% (*v*/*v*) acetonitrile and ultra-pure water, while phase B contained 55% (*v*/*v*) acetonitrile and ultra-pure water. Both phases were acidified with ortho-phosphoric acid to maintain a pH of 3.0. The total analysis time was 38 min, following the gradient program:

1.00–22.99 min: 95% phase A, 5% phase B.

23.00–27.99 min: 50% phase A, 50% phase B.

28.00–28.99 min: 80% phase A, 20% phase B.

29.00–42.00 min: 95% phase A, 5% phase B.

Detection wavelengths were set at 250 nm for phenolic acids and 370 nm for flavonols.

#### 4.4.3. Identification and Calculation

Phenolic compounds were identified through comparisons with retention times of pure standards: gallic, chlorogenic, caffeic, p-coumaric, sinapic, t-cinaminic acids, quercetin-3-O-rutinoside, kaempferol-3-O-glucoside, luteolin, quercetin, apigenin, and kaempferol (Sigma-Aldrich, Warsaw, Poland), each possessing 99.9% purity. Standard solutions were prepared from these pure compounds, with five injections performed for each standard. Chromatographic peak areas were recorded and calculated based on standard solution concentrations (Figures S1 and S2). From the obtained standard curves (Figures S3 and S4), mathematical equations were derived. Using these equations and dilution coefficients, the concentrations of individual phenolic compounds were determined.

### 4.5. Statistical Analysis

Statistical calculations were performed with the use of Statgraphics Centurion 15.2.11.0 software (StatPoint Technologies, Inc., Warranton, VA, USA). The PCA figures were made using XLStat (Microsoft Excel version 16.18). Results were compared using two-way analysis of variance (ANOVA) Sidak’s multiple comparisons test. Statistically significant results were marked and *p* value was reported for each significant pair of mean results.

## 5. Conclusions

Impact of organic and conventional farming on allergenic compounds: While the exact mechanism of profilin depletion is to be elucidated, organic farming is largely conducive to reducing the panallergenicity of wheat. Cereals exhibiting halved profilin content can be obtained without applying methods of genetic manipulation or external chemical induction. Despite a lower yield compared to conventional methods of agriculture, wheat grown organically becomes characterized by improved food safety and its functional profile for patients with hypersensitivities.

Impact of organic and conventional farming on bioactive compounds: Wheat was shown to contain a set of bioactive and possibly health-promoting polyphenols, including kaempferol, p-coumaric acid, and gallic acid. The distribution thereof was presented in detail throughout eight different varieties of wheat, either organic or conventional. The fact of differences reported between organic and conventional produce holds promise for the potential influence to be exerted by adopting specific cultivation conditions. The scale of reported differences, however, was not yet sufficient enough to conclude on the superiority of either of them.

## Figures and Tables

**Table 1:** The content of identified polyphenol (mg/100 g of grains) compounds in wheat samples from organic and conventional production.

Polyphenol	Cultivation System		*p*-Value
	Organic Wheat	Conventional Wheat	
gallic	21.21 ± 2.46 ^a^	20.58 ± 2.42 ^b^	<0.0001
chlorogenic	2.05 ± 0.2 ^a^	1.90 ± 0.23 ^b^	<0.0001
caffeic	3.82 ± 0.61 ^a^	3.61 ± 0.61 ^b^	<0.0001
p-coumaric	29.45 ± 4.09 ^a^	27.22 ± 3.89 ^b^	<0.0001
sinapic	2.46 ± 0.64 ^a^	2.19 ± 0.65 ^b^	<0.0001
t-cinaminic	2.62 ± 0.35 ^a^	2.55 ± 0.40 ^b^	0.0048
quercetin-3-*O*-rutinoside	3.23 ± 0.45 ^a^	2.95 ± 0.45 ^b^	<0.0001
kaempferol-3-*O*-glucoside	3.01 ± 0.58 ^a^	2.70 ± 0.56 ^b^	<0.0001
luteolin	0.63 ± 0.09 ^a^	0.56 ± 0.08 ^b^	<0.0001
quercetin	1.15 ± 0.17 ^a^	1.04 ± 0.18 ^b^	0.0003
apigenin	0.46 ± 0.10 ^b^	0.47 ± 0.08 ^a^	<0.0001
kaempferol	36.96 ± 2.67 ^b^	38.16 ± 3.14 ^a^	<0.0001

Data are presented as the mean ± SD with ANOVA *p*-value; means in rows followed by the different letters are significantly different at the 5% level of probability (*p* < 0.05); n = 24.

**Table 2:** The relationship between cultivation system and wheat varieties (in winter wheat) in the content of identified polyphenol (mg/100 g of grains) compounds.

Polyphenol	Organic Winter Wheat				Conventional Winter Wheat				*p*-Value
	Arkadia	Bamberka	Jantarka	Sailor	Arkadia	Bamberka	Jantarka	Sailor	
gallic	25.68 ± 0.17 ^a^	21.93 ± 0.24 ^c^	23.44 ± 0.17 ^ab^	22.17 ± 0.20 ^b^	25.00 ± 0.19 ^a^	21.67 ± 0.20 ^c^	22.90 ± 0.02 ^b^	20.81 ± 0.13 ^c^	0.003
chlorogenic	2.01 ± 0.04 ^b^	2.16 ± 0.03 ^b^	2.46 ± 0.04 ^a^	2.21 ± 0.05 ^b^	1.92 ± 0.01 ^c^	1.88 ± 0.01 ^c^	2.45 ± 0.03 ^a^	1.96 ± 0.04 ^c^	0.0001
caffeic	4.72 ± 0.03 ^a^	4.63 ± 0.05 ^a^	4.25 ± 0.01 ^a^	3.76 ± 0.03 ^a^	4.57 ± 0.01 ^a^	4.38 ± 0.04 ^a^	4.05 ± 0.12 ^a^	3.58 ± 0.02 ^a^	n.s.
p-coumaric	23.34 ± 0.29 ^b^	24.34 ± 0.026 ^b^	26.41 ± 0.14 ^ab^	32.02 ± 1.89 ^a^	21.53 ± 0.55 ^c^	21.58 ± 0.96 ^c^	25.80 ± 0.54 ^b^	26.92 ± 0.54 ^ab^	0.011
sinapic	1.57 ± 0.03 ^a^	1.67 ± 0.04 ^a^	2.17 ± 0.04 ^a^	2.02 ± 0.01 ^a^	1.35 ± 0.01 ^a^	1.40 ± 0.01 ^a^	1.87 ± 0.02 ^a^	1.69 ± 0.02 ^a^	n.s.
t-cinaminic	2.05 ± 0.02 ^c^	2.15 ± 0.03 ^c^	2.60 ± 0.01 ^a^	2.50 ± 0.02 ^b^	1.85 ± 0.03 ^c^	2.11 ± 0.02 ^c^	2.95 ± 0.01 ^a^	2.26 ± 0.06 ^b^	<0.0001
quercetin-3-*O*-rutinoside	2.81 ± 0.04 ^ab^	2.97 ± 0.01 ^a^	2.78 ± 0.04 ^b^	3.22 ± 0.04 ^a^	2.40 ± 0.04 ^c^	2.79 ± 0.04 ^b^	2.66 ± 0.06 ^c^	3.04 ± 0.01 ^a^	0.0001
kaempferol-3-*O*-glucoside	2.27 ± 0.08 ^b^	2.33 ± 0.04 ^b^	2.59 ± 0.03 ^b^	3.83 ± 0.11 ^a^	1.84 ± 0.05 ^c^	2.12 ± 0.02 ^c^	2.22 ± 0.03 ^b^	3.33 ± 0.03 ^a^	0.015
luteolin	0.55 ± 0.01 ^a^	0.54 ± 0.02 ^a^	0.56 ± 0.01 ^a^	0.58 ± 0.01 ^a^	0.48 ± 0.01 ^b^	0.46 ± 0.01 ^b^	0.49 ± 0.01 ^b^	0.55 ± 0.01 ^a^	0.048
quercetin	0.91 ± 0.02 ^a^	0.98 ± 0.02 ^a^	1.04 ± 0.01 ^a^	1.08 ± 0.01 ^a^	0.78 ± 0.01 ^b^	0.87 ± 0.02 ^b^	0.97 ± 0.01 ^a^	1.03 ± 0.02 ^a^	0.034
apigenin	0.62 ± 0.005 ^a^	0.57 ± 0.012 ^ab^	0.50 ± 0.003 ^b^	0.52 ± 0.004 ^b^	0.59 ± 0.004 ^a^	0.55 ± 0.001 ^b^	0.52 ± 0.006 ^b^	0.55 ± 0.003 ^b^	<0.0001
kaempferol	38.01 ± 0.09 ^b^	39.89 ± 0.4 ^b^	41.05 ± 0.11 ^a^	38.40 ± 0.88 ^b^	38.15 ± 0.44 ^b^	43.35 ± 0.26 ^a^	42.79 ± 0.23 ^a^	38.35 ± 0.45 ^b^	0.0001

Data are presented as the mean ± SD with ANOVA *p*-value; means in rows followed by the different letters are significantly different at the 5% level of probability (*p* < 0.05); n = 3; n.s. not significant statistically.

**Table 3:** The relationship between cultivation system and wheat varieties (in spring wheat) in the content of identified polyphenol (mg/100 g of grains) compounds.

Polyphenol	Organic Winter Wheat				Conventional Winter Wheat				*p*-Value
	Ostka Smolicka	Waluta	Kandela	Izera	Ostka Smolicka	Waluta	Kandela	Izera	
gallic	18.42 ± 0.20 ^a^	18.00 ± 0.21 ^a^	19.30 ± 0.15 ^a^	20.78 ± 0.16 ^a^	17.87 ± 0.16 ^a^	17.30 ± 0.28 ^a^	19.25 ± 0.17 ^a^	19.82 ± 0.33 ^a^	n.s.
chlorogenic	1.93 ± 0.03 ^a^	1.86 ± 0.03 ^b^	1.82 ± 0.02 ^b^	1.94 ± 0.05 ^a^	1.66 ± 0.05 ^c^	1.79 ± 0.01 ^b^	1.70 ± 0.06 ^c^	1.79 ± 0.07 ^b^	0.013
caffeic	3.19 ± 0.03 ^b^	3.67 ± 0.07 ^a^	3.19 ± 0.01 ^b^	3.10 ± 0.03 ^b^	3.03 ± 0.08 ^b^	3.36 ± 0.02 ^b^	3.05 ± 0.01 ^b^	2.85 ± 0.01 ^c^	0.047
p-coumaric	29.37 ± 0.32 ^c^	32.14 ± 0.19 ^a^	33.04 ± 0.12 ^a^	34.95 ± 0.36 ^a^	27.84 ± 0.15 ^c^	31.10 ± 0.36 ^b^	31.40 ± 0.21 ^b^	31.61 ± 0.59 ^b^	0.0007
sinapic	2.88 ± 0.04 ^b^	2.98 ± 0.04 ^b^	3.17 ± 0.03 ^a^	3.24 ± 0.03 ^a^	2.71 ± 0.02 ^c^	2.59 ± 0.01 ^c^	2.80 ± 0.02 ^b^	3.11 ± 0.02 ^a^	<0.0001
t-cinaminic	2.78 ± 0.08 ^c^	2.80 ± 0.01 ^b^	3.13 ± 0.05 ^a^	2.96 ± 0.01 ^a^	2.81 ± 0.02 ^b^	2.63 ± 0.03 ^c^	2.96 ± 0.01 ^a^	2.82 ± 0.02 ^b^	0.003
quercetin-3-O-rutinoside	2.76 ± 0.01 ^b^	3.58 ± 0.06 ^a^	3.73 ± 0.05 ^a^	3.96 ± 0.02 ^a^	2.38 ± 0.03 ^b^	3.15 ± 0.04 ^a^	3.54 ± 0.12 ^a^	3.65 ± 0.03 ^a^	0.035
kaempferol-3-O-glucoside	2.86 ± 0.05 ^b^	3.01 ± 0.01 ^b^	3.35 ± 0.05 ^ab^	3.85 ± 0.01 ^a^	2.68 ± 0.04 ^c^	2.87 ± 0.05 ^b^	2.96 ± 0.04 ^c^	3.55 ± 0.10 ^a^	0.011
luteolin	0.62 ± 0.02 ^a^	0.66 ± 0.01 ^a^	0.73 ± 0.06 ^a^	0.81 ± 0.02 ^a^	0.52 ± 0.02 ^a^	0.62 ± 0.01 ^a^	0.63 ± 0.01 ^a^	0.71 ± 0.02 ^a^	n.s.
quercetin	1.17 ± 0.02 ^b^	1.24 ± 0.02 ^b^	1.36 ± 0.02 ^a^	1.42 ± 0.02 ^a^	1.03 ± 0.02 ^c^	1.12 ± 0.02 ^c^	1.17 ± 0.01 ^c^	1.38 ± 0.03 ^a^	0.001
apigenin	0.37 ± 0.01 ^c^	0.36 ± 0.01 ^c^	0.39 ± 0.01 ^b^	0.38 ± 0.01 ^b^	0.41 ± 0.01 ^a^	0.38 ± 0.01 ^b^	0.40 ± 0.01 ^a^	0.39 ± 0.01 ^ab^	0.027
kaempferol	34.86 ± 0.23 ^a^	33.30 ± 0.26 ^a^	34.01 ± 0.30 ^a^	36.14 ± 0.22 ^a^	36.12 ± 0.25 ^a^	34.27 ± 0.37 ^a^	35.20 ± 0.17 ^a^	37.02 ± 0.71 ^a^	n.s.

Data are presented as the mean ± SD with ANOVA *p*-value; means in rows followed by the different letters are significantly different at the 5% level of probability (*p* < 0.05); n = 3; n.s. not significant statistically.

**Table 4:** Management of spring wheat and winter wheat in different crop production systems.

	Crop Production System	
Specification	Organic	Conventional
Crop rotation	potato spring wheat + clovers and grasses undersown clovers and grasses (1st year) clovers and grasses (2nd year) winter wheat + catch crop	winter rape winter wheat spring wheat
Organic fertilization	compost (30 t∙ha^−1^) to potato; catch crop	rape straw winter wheat straw
Mineral fertilization NPK (kg∙ha^−1^)	According to the results of soil analysis and crop needs, natural P + K fertilizers (42 + 60) in 2013	Spring wheat: N (110) + P (35) + K (70) Winter wheat: N (140) + P (60) + K (80)
Herbicides	-	2 treatments (see Table 2)
Fungicides	-	2 treatments (see Table 2)
Insecticides	-	2 treatments (see Table 2)

**Table 5:** Plant protection products used in different crop production systems in spring wheat and winter wheat.

Crop Production System	Plant Protection Products		
	Herbicides	Fungicides	Insecticides
Organic	-	-	-
Conventional	Mustang Forte 195SE 0.8 L ha^−1^	Tilt Turbo 575EC 1.0 L ha^−1^	Decis Mega 0.125 L ha^−1^
	Axial 100EC 0.4 L ha^−1^ + Agritox Turbo 750SL 1.0 L ha^−1^	Menara 410EC 0.4 L ha^−1^ + Amistar 250 SC 0.6 L ha^−1^	Furry 100EW 0.1 L ha^−1^
	Mustang Forte 195 SE: aminopyralid—10 g·L^−1^, florasulam—5 g·L^−1^, 2,4D—180 g·L^−1^; Axial 100EC: pinoxaden—100 g·L^−1^; Agritox Turbo 750SL: MCPA—660 g·L^−1^, dicamb—90 g·L^−1^	Tilt Turbo 575EC: propiconazole—125 g·L^−1^, fenpropidine—450 g·L^−1^; Menara 410EC: propiconazole 250 g·L^−1^, Amistar 250SC: azoxystrobin 250 g·L^−1^	Decis Mega: deltamethrin—50 g·L^−1^; Furry 100EW: zeta-cypermethrin —100 g·L^−1^

## Data Availability

Data are contained within the article and Supplementary Materials.

## Supplementary Materials

The following supporting information can be downloaded at: https://www.mdpi.com/article/10.3390/molecules30061313/s1, Figure S1: The example of chromatogram from phenolic separation for organic wheat (1) gallic acid, (2) caffeic acid, (3) chlorogenic acid, (4) sinapic acid, (6) quercetin-3-O-rutinoside, (7) kaempferol-3-O-glucoside, (8) t-cinaminic acid, (9) quercetin, (10) apigenin, (11) kaempferol, (12) luteolin; Figure S2: The example of chromatogram from phenolic separation for conventional wheat (1) gallic acid, (2) caffeic acid, (3) chlorogenic acid, (4) sinapic acid, (6) quercetin-3-O-rutinoside, (7) kaempferol-3-O-glucoside, (8) t-cinaminic acid, (9) quercetin, (10) apigenin, (11) kaempferol, (12) luteolin; Figure S3: Standard curves for phenolic acids identified in examined wheats cultivars; Figure S4: Standard curves for flavonoids identified in examined wheats cultivars.

## References

[1] Moysiadis V., Sarigiannidis P., Vitsas V., Khelifi A. (2021). Smart Farming in Europe. Comput. Sci. Rev..

[2] Krasilnikov P., Taboada M.A., Amanullah (2022). Fertilizer Use, Soil Health and Agricultural Sustainability. Agriculture.

[3] Xie E., Zhao Y., Li H., Shi X., Lu F., Zhang X., Peng Y. (2019). Spatio-temporal changes of cropland soil pH in a rapidly industrializing region in the Yangtze River Delta of China, 1980–2015. Agric. Ecosyst. Environ..

[4] Dhankhar N., Kumar J. (2023). Impact of increasing pesticides and fertilizers on human health: A review. Mater. Today Proc..

[5] Zhang X., Li J., Shao L., Qin F., Yang J., Gu H., Zhai P., Pan X. (2023). Effects of organic fertilizers on yield, soil physico-chemical property, soil microbial community diversity and structure of Brassica rapa var. Chinensis. Front. Microbiol..

[6] Yuan D., Hu Y., Jia S., Li W., Zamanian K., Han J., Huang F., Zhao X. (2023). Microbial Properties Depending on Fertilization Regime in Agricultural Soils with Different Texture and Climate Conditions: A Meta-Analysis. Agronomy.

[7] Frattini N., Pulido Carrasquero A., Pronsato L., Milanesi L., Vasconsuelo A. (2023). Effects of common fertilizers on the soil ecosystem. Bull. Natl. Res. Cent..

[8] Wyer K.E., Kelleghan D.B., Blanes-Vidal V., Schauberger G., Curran T.P. (2022). Ammonia emissions from agriculture and their contribution to fine particulate matter: A review of implications for human health. J. Environ. Manag..

[9] Li A., Shi Z., Yin Y., Fan Y., Zhang Z., Tian X., Yang Y., Pan L. (2023). Excessive use of chemical fertilizers in catchment areas raises the seasonal pH in natural freshwater lakes of the subtropical monsoon climate region. Ecol. Indic..

[10] Tudi M., Daniel Ruan H., Wang L., Lyu J., Sadler R., Connell D., Chu C., Phung D.T. (2021). Agriculture Development, Pesticide Application and Its Impact on the Environment. Int. J. Environ. Res. Public Health.

[11] Pathak V.M., Verma V.K., Rawat B.S., Kaur B., Babu N., Sharma A., Dewali S., Yadav M., Kumari R., Singh S. (2022). Current status of pesticide effects on environment, human health and it’s eco-friendly management as bioremediation: A comprehensive review. Front. Microbiol..

[12] Riedo J., Wächter D., Gubler A., Wettstein F.E., Meuli R.G., Bucheli T.D. (2023). Pesticide residues in agricultural soils in light of their on-farm application history. Environ. Pollut..

[13] Bras A., Roy A., Heckel D.G., Anderson P., Karlsson Green K. (2022). Pesticide resistance in arthropods: Ecology matters too. Ecol. Lett..

[14] Thia J.A., Maino J., Kelly A., Hoffmann A.A., Umina P.A. (2023). Expanding risk predictions of pesticide resistance evolution in arthropod pests with a proxy for selection pressure. J. Pest Sci..

[15] Scorza F.A., Beltramim L., Bombardi L.M. (2023). Pesticide exposure and human health: Toxic legacy. Clinics.

[16] Penuelas J., Coello F., Sardans J. (2023). A better use of fertilizers is needed for global food security and environmental sustainability. Agric. Food Secur..

[17] Wei B., Yang Z. (2022). Government promotion, social networks and farmers’ adoption behavior of ecological farming technology. Chin. J. Eco-Agric..

[18] Rega C., Thompson B., Niedermayr A., Desjeux Y., Kantelhardt J., D’Alberto R., Gouta P., Konstantidelli V., Schaller L., Latruffe L. (2022). Uptake of Ecological Farming Practices by EU Farms: A Pan-European Typology. EuroChoices.

[19] Constantin M., Deaconu M.E., Petrescu I.-E., Istudor M., Tărăşilă G.A. (2022). A review on the competitiveness and performance of ecological, organic and regenerative agricultural systems. Proc. Int. Conf. Bus. Excell..

[20] Knapp S., van der Heijden M.G.A. (2018). A global meta-analysis of yield stability in organic and conservation agriculture. Nat. Commun..

[21] Smith O.M., Cohen A.L., Rieser C.J., Davis A.G., Taylor J.M., Adesanya A.W., Jones M.S., Meier A.R., Reganold J.P., Orpet R.J. (2019). Organic Farming Provides Reliable Environmental Benefits but Increases Variability in Crop Yields: A Global Meta-Analysis. Front. Sustain. Food Syst..

[22] Cakmakci S., Cakmakci R. (2023). Quality and Nutritional Parameters of Food in Agri-Food Production Systems. Foods.

[23] Rej A., Aziz I., Sanders D.S. (2020). Coeliac disease and noncoeliac wheat or gluten sensitivity. J. Intern. Med..

[24] Spector Cohen I., Day A., Shaoul R. (2020). Should the Glu Be Ten or Twenty? An Update on the Ongoing Debate on Gluten Safety Limits for Patients with Celiac Disease. Gastrointest. Disord..

[25] Matsuo H., Yokooji T., Taogoshi T. (2015). Common food allergens and their IgE-binding epitopes. Allergol. Int..

[26] García-Ramírez B., Mares-Mejía I., Rodríguez-Hernández A., Cano-Sánchez P., Torres-Larios A., Ortega E., Rodríguez-Romero A. (2022). A native IgE in complex with profilin provides insights into allergen recognition and cross-reactivity. Commun. Biol..

[27] Alvarado M.I., Jimeno L., De La Torre F., Boissy P., Rivas B., Lázaro M.J., Barber D. (2014). Profilin as a severe food allergen in allergic patients overexposed to grass pollen. Allergy.

[28] Yigezu Y.A., Mugera A., El-Shater T., Aw-Hassan A., Piggin C., Haddad A., Khalil Y., Loss S. (2018). Enhancing adoption of agricultural technologies requiring high initial investment among smallholders. Technol. Forecast. Soc. Change.

[29] Colivicchi I. (2022). From Industrial Agriculture Towards Ecological Farming-Necessity or Opportunity.

[30] Popa M.E., Mitelut A.C., Popa E.E., Stan A., Popa V.I. (2019). Organic foods contribution to nutritional quality and value. Trends Food Sci. Technol..

[31] Hurtado-Barroso S., Tresserra-Rimbau A., Vallverdu-Queralt A., Lamuela-Raventos R.M. (2019). Organic food and the impact on human health. Crit. Rev. Food Sci. Nutr..

[32] Bhagavathula A.S., Vidyasagar K., Khubchandani J. (2022). Organic Food Consumption and Risk of Obesity: A Systematic Review and Meta-Analysis. Healthcare.

[33] Gosling C.J., Goncalves A., Ehrminger M., Valliant R. (2021). Association of organic food consumption with obesity in a nationally representative sample. Br. J. Nutr..

[34] Rahman A., Baharlouei P., Koh E.H.Y., Pirvu D.G., Rehmani R., Arcos M., Puri S. (2024). A Comprehensive Analysis of Organic Food: Evaluating Nutritional Value and Impact on Human Health. Foods.

[35] Mitura K., Cacak-Pietrzak G., Feledyn-Szewczyk B., Szablewski T., Studnicki M. (2023). Yield and Grain Quality of Common Wheat (*Triticum aestivum* L.) Depending on the Different Farming Systems (Organic vs. Integrated vs. Conventional). Plants.

[36] Krejčířová L., Capouchová I., Petr J., Bicanová E., Faměra O. (2007). The effect of organic and conventional growing systems on quality and storage protein composition of winter wheat. Plant Soil Environ..

[37] Rozbicki J., Ceglińska A., Gozdowski D., Jakubczak M., Cacak-Pietrzak G., Mądry W., Golba J., Piechociński M., Sobczyński G., Studnicki M. (2015). Influence of the cultivar, environment and management on the grain yield and bread-making quality in winter wheat. J. Cereal Sci..

[38] Sobolewska M., Stankowski S. (2017). The influence of farming systems on the technological quality of grain and flour cultivars of winter wheat. Folia Pomeranae Univ. Technol. Stetin. Agric. Aliment. Piscaria Zootech..

[39] Zhou E., Xue X., Xu H., Zhao L., Wu L., Li Q. (2023). Effects of covalent conjugation with quercetin and its glycosides on the structure and allergenicity of Bra c p from bee pollen. Food Chem..

[40] Davey R.J., Moens P.D. (2020). Profilin: Many facets of a small protein. Biophys. Rev..

[41] Suanno C., Aloisi I., Parrotta L., Fernández-González D., Del Duca S. (2021). Allergenic risk assessment of urban parks: Towards a standard index. Environ. Res..

[42] Jeon Y.H. (2020). Pollen-food allergy syndrome in children. Clin. Exp. Pediatr..

[43] Akinfenwa O., Huang H.J., Linhart B., Focke-Tejkl M., Vrtala S., Poroshina A., Nikonova A., Khaitov M., Campion N.J., Eckl-Dorna J. (2021). Preventive Administration of Non-Allergenic Bet v 1 Peptides Reduces Allergic Sensitization to Major Birch Pollen Allergen, Bet v 1. Front. Immunol..

[44] Olivieri M., Spiteri G., Brandi J., Cecconi D., Fusi M., Zanoni G., Rizzi C. (2022). Glucose/Ribitol Dehydrogenase and 16.9 kDa Class I Heat Shock Protein 1 as Novel Wheat Allergens in Baker’s Respiratory Allergy. Molecules.

[45] Costantino A., Aversano G.M., Lasagni G., Smania V., Doneda L., Vecchi M., Roncoroni L., Pastorello E.A., Elli L. (2022). Diagnostic management of patients reporting symptoms after wheat ingestion. Front. Nutr..

[46] Zhang Y., Che H., Li C., Jin T. (2023). Food Allergens of Plant Origin. Foods.

[47] Słowianek M., Skorupa M., Hallmann E., Rembiałkowska E., Leszczyńska J. (2016). Allergenic Potential of Tomatoes Cultivated in Organic and Conventional Systems. Plant Foods Hum. Nutr..

[48] Hallmann E., Rozpara E., Slowianek M., Leszczynska J. (2019). The effect of organic and conventional farm management on the allergenic potency and bioactive compounds status of apricots (*Prunus armeniaca* L.). Food Chem..

[49] Aninowski M., Leszczyńska J. (2019). The determination of potentially allergenicity of selected herbs. Biotechnol. Food Sci..

[50] Hallmann E., Ponder A., Aninowski M., Narangerel T., Leszczyńska J. (2020). The Interaction between Antioxidants Content and Allergenic Potency of Different Raspberry Cultivars. Antioxidants.

[51] Alrumaihi F., Almatroodi S.A., Alharbi H.O.A., Alwanian W.M., Alharbi F.A., Almatroudi A., Rahmani A.H. (2024). Pharmacological Potential of Kaempferol, a Flavonoid in the Management of Pathogenesis via Modulation of Inflammation and Other Biological Activities. Molecules.

[52] Tehami W., Nani A., Khan N.A., Hichami A. (2023). New Insights Into the Anticancer Effects of p-Coumaric Acid: Focus on Colorectal Cancer. Dose Response.

[53] Pei K., Ou J., Huang J., Ou S. (2016). p-Coumaric acid and its conjugates: Dietary sources, pharmacokinetic properties and biological activities. J. Sci. Food Agric..

[54] Yu X.D., Zhang D., Xiao C.L., Zhou Y., Li X., Wang L., He Z., Reilly J., Xiao Z.Y., Shu X. (2022). P-Coumaric Acid Reverses Depression-Like Behavior and Memory Deficit Via Inhibiting AGE-RAGE-Mediated Neuroinflammation. Cells.

[55] Cao Y., Chen H., Tan Y., Yu X.-D., Xiao C., Li Y., Reilly J., He Z., Shu X. (2024). Protection of p-Coumaric acid against chronic stress-induced neurobehavioral deficits in mice via activating the PKA-CREB-BDNF pathway. Physiol. Behav..

[56] Wang L., You X., Dai C., Fang Y., Wu J. (2022). Development of poly(p-coumaric acid) as a self-anticancer nanocarrier for efficient and biosafe cancer therapy. Biomater. Sci..

[57] Hadidi M., Linan-Atero R., Tarahi M., Christodoulou M.C., Aghababaei F. (2024). The Potential Health Benefits of Gallic Acid: Therapeutic and Food Applications. Antioxidants.

[58] Holghoomi R., Kiani M.H., Rahdar A., Hashemi S.M., Romanholo Ferreira L.F., Fathi-karkan S. (2024). Nanoparticle-delivered gallic acid: A new frontier in cancer therapy. J. Drug Deliv. Sci. Technol..

[59] Behera P.K., Devi S., Mittal N. (2023). Therapeutic potential of gallic acid in obesity: Considerable shift!. Obes. Med..

[60] Bhuia M.S., Rahaman M.M., Islam T., Bappi M.H., Sikder M.I., Hossain K.N., Akter F., Al Shamsh Prottay A., Rokonuzzman M., Gürer E.S. (2023). Neurobiological effects of gallic acid: Current perspectives. Chin. Med..

[61] Kowalska I., Soluch A., Mołdoch J., Jończyk K. (2025). The Effect of Farming Systems and Cultivars on the Qualitative and Quantitative Composition of Bioactive Compounds in Winter Wheat (*Triticum aestivum* L.). Molecules.

[62] Bartos A., Majak I., Leszczynska J. (2024). Detection of Bet v 1 Homologous Proteins and Plant Profilins by Indirect ELISA. Methods Mol. Biol..

