# Supplementary material for: Polyphenolic and Immunometric Profiling of Wheat Varieties: Impact of Organic and Conventional Farming on Allergenic and Bioactive Compounds
Source: Molecules. 2025 Mar 14;30(6):1313. doi: 10.3390/molecules30061313 (PMC11944287; doi:10.3390/molecules30061313)
Supplement: Supplementary file 1 [file molecules-30-01313-s001.zip › molecules-3471273-supplementary.pdf]

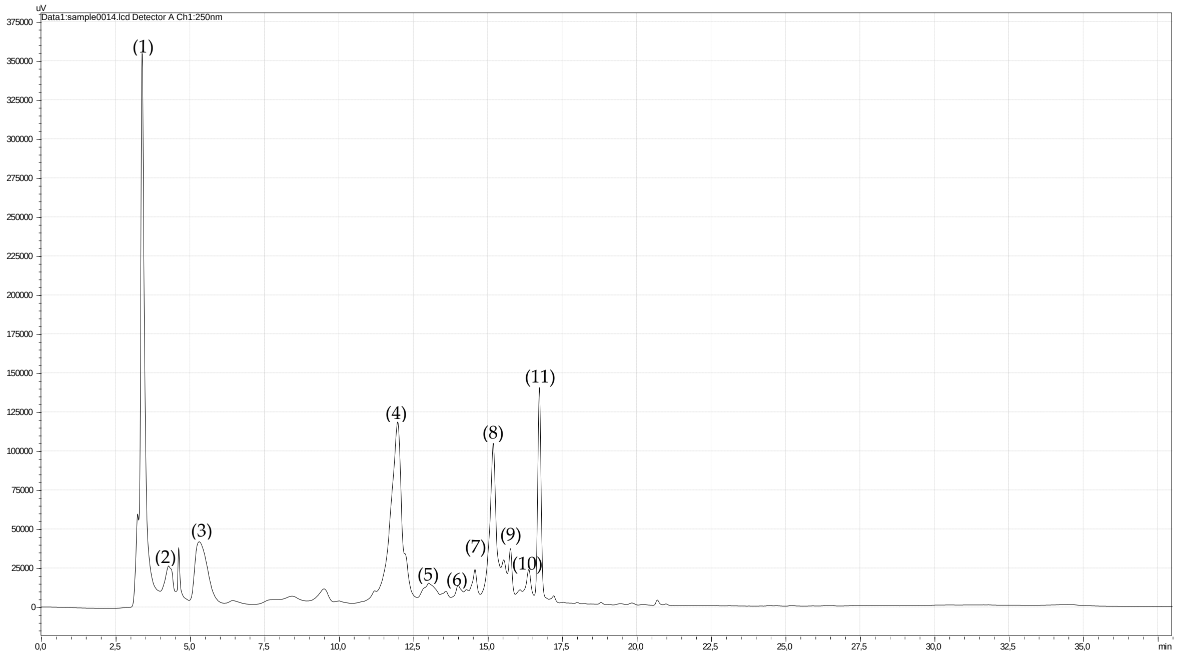

Figure S1. The example of chromatogram from phenolic separation for organic wheat (1) gallic acid, (2) caffeic acid, (3) chlorogenic acid, (4) sinapic acid, (6) quercetin-3-O-rutinoside, (7) kaempferol-3-O-glucoside, (8) t-cinaminic acid, (9) quercetin, (10) apigenin, (11) kaempferol, (12) luteolin.

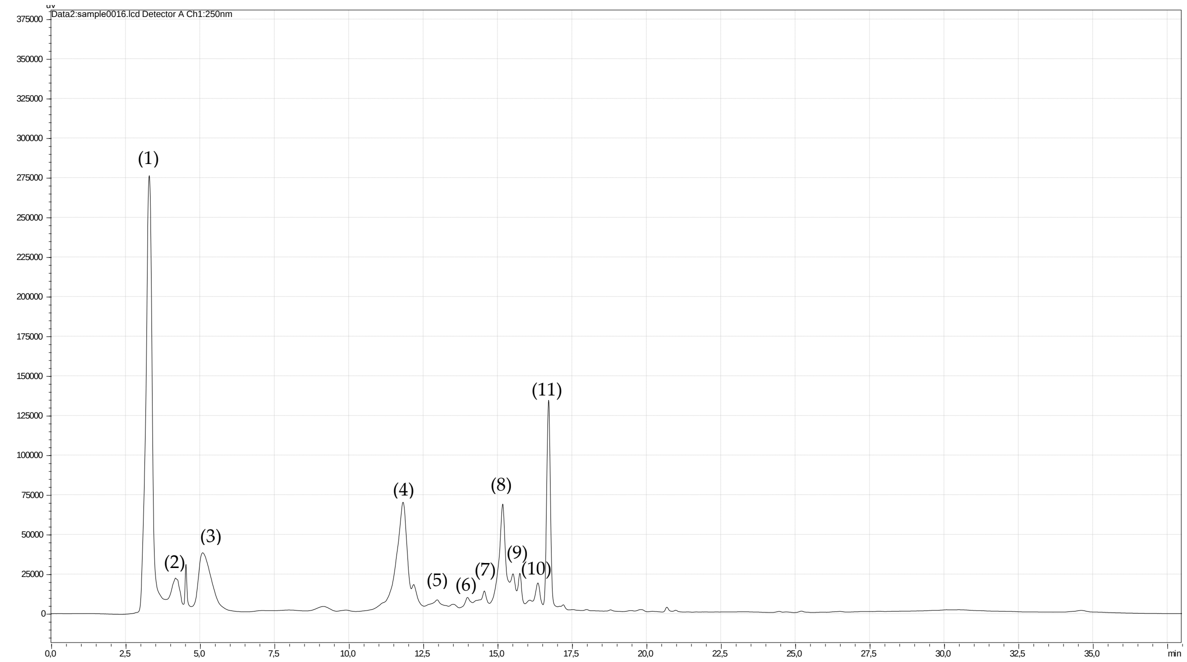

Figure S2. The example of chromatogram from phenolic separation for conventional wheat (1) gallic acid, (2) caffeic acid, (3) chlorogenic acid, (4) sinapic acid, (6) quercetin-3-O-rutinoside, (7) kaempferol-3-O-glucoside, (8) t-cinaminic acid, (9) quercetin, (10) apigenin, (11) kaempferol, (12) luteolin.

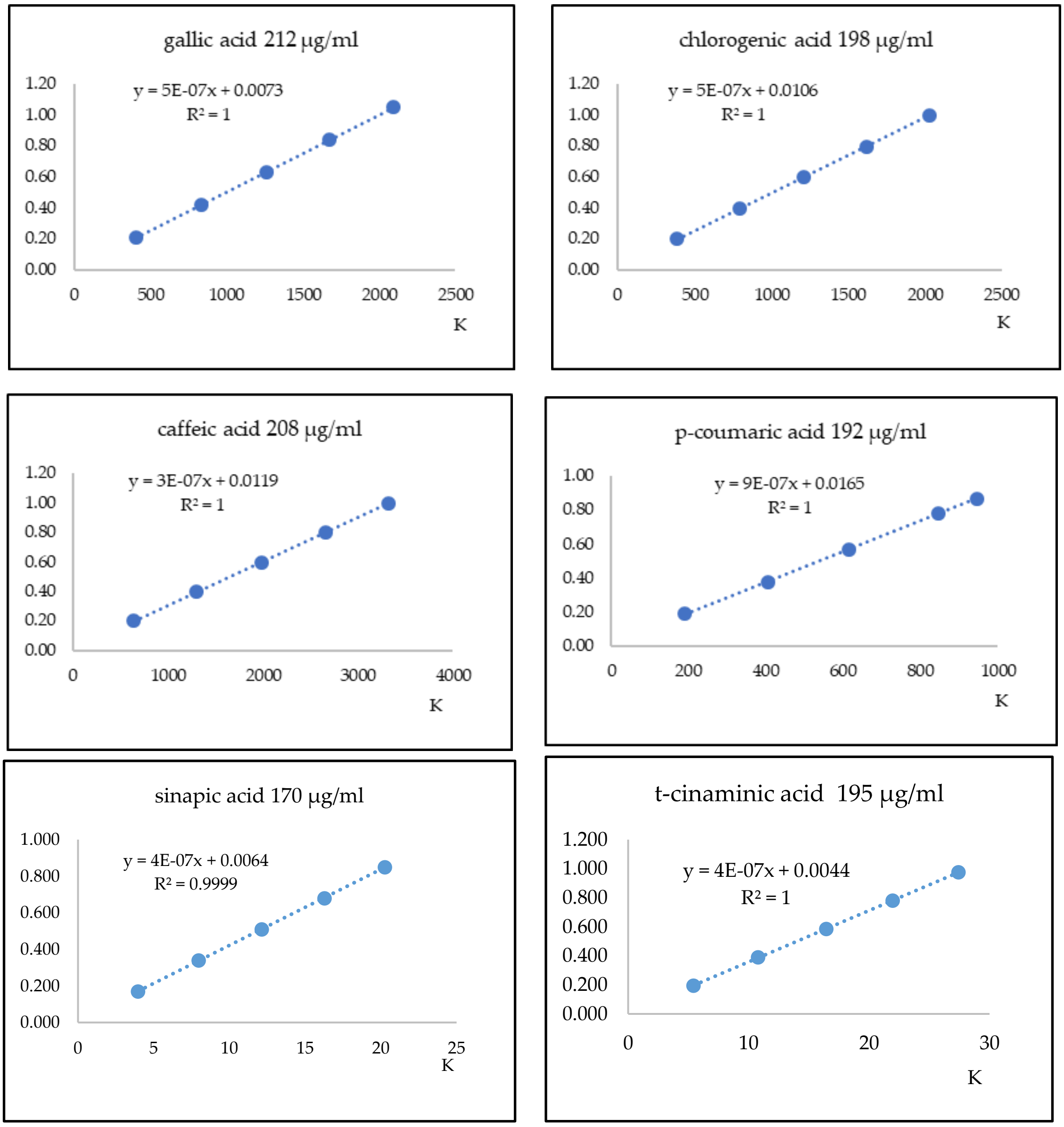

Figure 3S Standard curves for phenolic acids identified in examined wheats cultivars

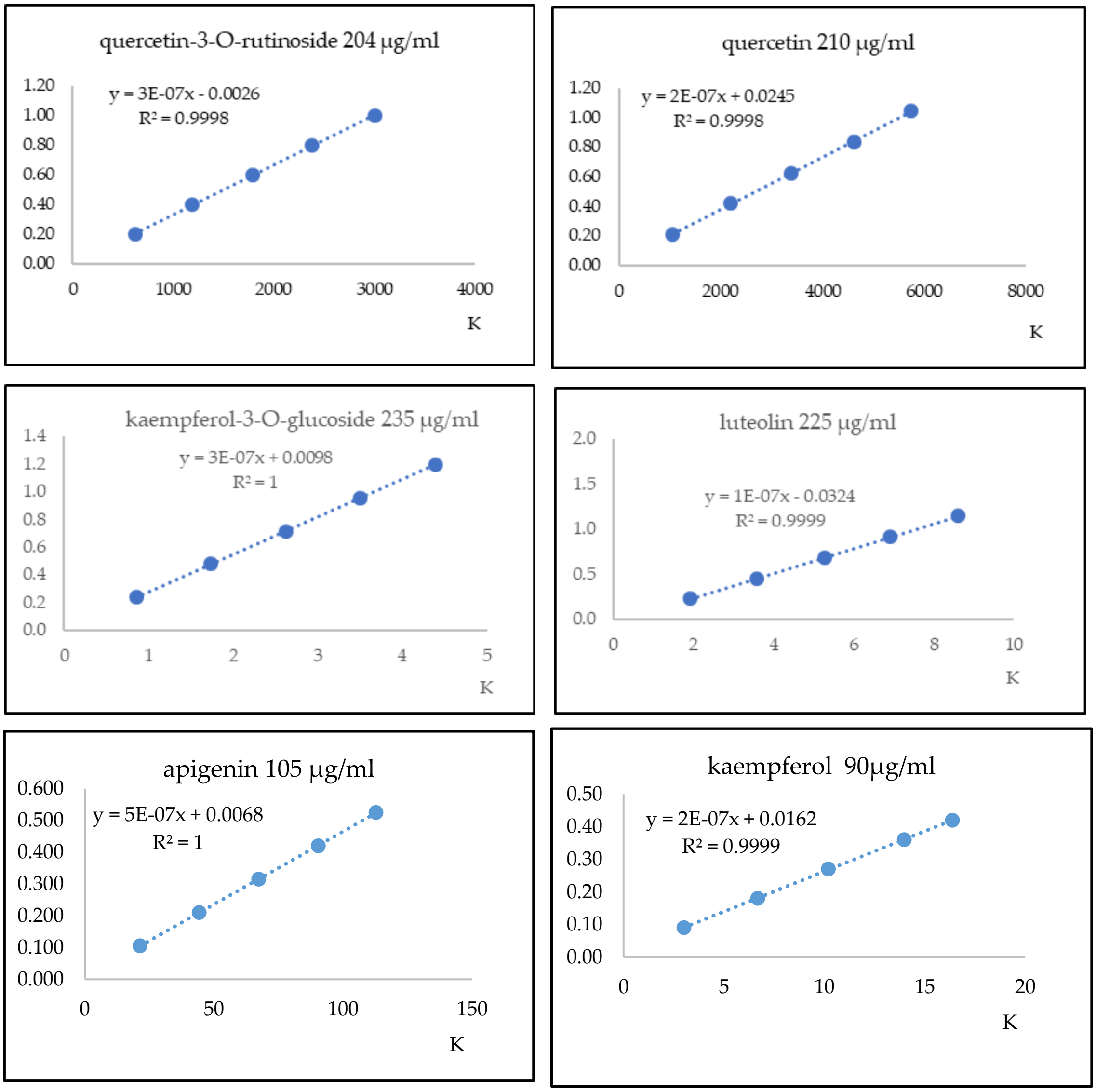

Figure S4. Standard curves for flavonoids identified in examined wheats cultivars
